# Advancements in SARS-CoV-2 Testing: Enhancing Accessibility through Machine Learning-Enhanced Biosensors

**DOI:** 10.3390/mi14081518

**Published:** 2023-07-28

**Authors:** Antonios Georgas, Konstantinos Georgas, Evangelos Hristoforou

**Affiliations:** School of Electrical and Computer Engineering, National Technical University of Athens, 15780 Athens, Greece; kostasgeo@biomed.ntua.gr (K.G.); hristoforou@ece.ntua.gr (E.H.)

**Keywords:** COVID-19, sensors, biosensors, machine learning, deep learning, contrastive learning, artificial intelligence

## Abstract

The COVID-19 pandemic highlighted the importance of widespread testing for SARS-CoV-2, leading to the development of various new testing methods. However, traditional invasive sampling methods can be uncomfortable and even painful, creating barriers to testing accessibility. In this article, we explore how machine learning-enhanced biosensors can enable non-invasive sampling for SARS-CoV-2 testing, revolutionizing the way we detect and monitor the virus. By detecting and measuring specific biomarkers in body fluids or other samples, these biosensors can provide accurate and accessible testing options that do not require invasive procedures. We provide examples of how these biosensors can be used for non-invasive SARS-CoV-2 testing, such as saliva-based testing. We also discuss the potential impact of non-invasive testing on accessibility and accuracy of testing. Finally, we discuss potential limitations or biases associated with the machine learning algorithms used to improve the biosensors and explore future directions in the field of machine learning-enhanced biosensors for SARS-CoV-2 testing, considering their potential impact on global healthcare and disease control.

## 1. Introduction

Since the emergence of the COVID-19 pandemic, researchers worldwide continued working tirelessly to develop and improve testing methods in order to meet the urgent need for widespread and accurate detection of SARS-CoV-2. Early detection of the virus is crucial for controlling its spread, as it enables prompt identification and isolation of infected individuals. This is especially important given the highly contagious nature of the virus and the potential for asymptomatic transmission [1]. Testing is also essential for tracking the prevalence of the disease and informing public health policies [2].

However, traditional invasive sampling procedures for SARS-CoV-2 testing, such as nasopharyngeal swabs, can be uncomfortable and even painful for patients [3,4,5]. This can lead to reluctance to get tested, which can, in turn, lead to delayed diagnosis and increased transmission [6]. Moreover, the need for trained healthcare professionals to perform these invasive procedures can add additional strain to healthcare systems that are already overwhelmed with the demands of the pandemic [7,8].

Non-invasive methods of SARS-CoV-2 testing, such as the use of saliva samples [9], have the potential to address these challenges and improve testing accessibility [10]. Non-invasive testing methods can be more convenient and less painful for patients, reducing barriers to testing. Additionally, non-invasive methods can be performed by individuals themselves, reducing the need for trained healthcare professionals to perform testing procedures [11].

The involvement of artificial intelligence (such as machine learning and deep learning) in medical diagnosis [12] may be a key point in the fight against COVID-19 [13,14]. One promising approach to non-invasive SARS-CoV-2 testing involves the use of machine learning-enhanced biosensors. These biosensors can detect and measure specific biomarkers in saliva, providing accurate and accessible testing options that do not require invasive procedures. By leveraging machine learning algorithms, these biosensors can also provide real-time results and improve the accuracy of testing [15].

In this article, we will explore the potential of machine learning-enhanced biosensors for non-invasive SARS-CoV-2 testing. We will discuss the limitations of traditional invasive sampling procedures for SARS-CoV-2 testing, and then explore how machine learning-enhanced biosensors can enable non-invasive sampling. We will also provide examples of how these biosensors can be used for non-invasive SARS-CoV-2 testing, such as saliva-based testing. Finally, we will discuss the potential impact of non-invasive testing on testing accessibility and accuracy and consider the future directions in the field of machine learning-enhanced biosensors for SARS-CoV-2 testing. In this way, this review aims to provide valuable insights into the cutting-edge integration of machine learning with biosensing technology, paving the way for more accessible, sensitive, and efficient SARS-CoV-2 testing methods that can greatly contribute to global healthcare and disease control efforts.

## 2. The Current State of SARS-CoV-2 Testing

### 2.1. Overview of Current Invasive Testing Methods

The current gold standard for SARS-CoV-2 testing is the reverse transcription polymerase chain reaction (RT-PCR) test, which detects viral RNA. This method involves collecting a sample from the patient’s respiratory tract, usually via a nasopharyngeal swab or an oropharyngeal swab, and then sending the sample to a laboratory for analysis. While the RT-PCR test is highly accurate, it has some limitations. Firstly, it is time-consuming, as the samples need to be transported to a laboratory and analyzed using specialized equipment. Secondly, the test can be expensive, particularly in low-resource settings where the necessary equipment may not be readily available [16]. A recent systematic review emphasized that while RT-PCR remains the gold-standard method for COVID-19 diagnosis, its limitations prompted the exploration of alternative methods [17].

To address these limitations, rapid antigen tests, such as lateral flow assays [18], were developed for SARS-CoV-2 testing. These tests are less sensitive and specific compared to RT-PCR testing, but they provide results within minutes and can be performed outside of a laboratory setting. Lateral flow assays are based on the detection of viral proteins, and they involve collecting a sample from the patient’s respiratory tract, usually via a nasopharyngeal swab or an anterior nasal swab. The swab is then inserted into a tube containing a reagent, which reacts with the viral proteins in the sample. The resulting mixture is then applied to a test strip, which contains antibodies that can bind to the viral proteins. If the virus is present in the sample, a visible line appears on the test strip, indicating a positive result. While lateral flow assays are faster and cheaper than RT-PCR testing, they are less accurate and have a higher risk of false negatives [19].

As for serological testing, which is the utilization of tests that detect antibodies produced by the body’s immune system in response to an infection by SARS-CoV-2 [20], their utilization in SARS-CoV-2 testing sparked widespread discussion at various levels [21], offering potential for mass screening. However, there are various challenges and limitations, such as the varying performance characteristics of commercially available tests and the uncertainty regarding protective immunity. Some major issues are their weakness when it comes to accurately reflecting the immune response to the evolving SARS-CoV-2 variants due to the substantial mutations in the viral genome, leading to potential underestimation of anti-SARS-CoV-2 antibodies and rendering some tests inadequate in detecting antibodies against recent circulating variants. Additionally, the progressive waning of natural and vaccine-induced immunity poses a challenge, as anti-SARS-CoV-2 antibodies become undetectable over time, while cellular immunity remains protective [22].

In addition to RT-PCR and lateral flow assays, biosensors are also being developed for SARS-CoV-2 testing [23,24,25]. Biosensors are analytical devices that use biological or biochemical reactions to detect specific molecules or compounds. They have the potential to provide highly sensitive and specific detection of viral particles, even in low concentrations, without the need for extensive sample processing or specialized laboratory equipment. Biosensors can also be designed to be highly specific to SARS-CoV-2, reducing the risk of false positives or cross-reactivity with other viruses. Several biosensors are also being developed for point of care (PoC) treatment, thus making it easier for the patient to perform the test even by himself/herself [26,27]. While biosensors are still in the development stage, they hold great promise for improving the accuracy and speed of SARS-CoV-2 testing, particularly in resource-limited settings where access to laboratory facilities may be limited [25].

### 2.2. Limitations of Invasive Testing Methods

While invasive testing methods can provide accurate results, they have several limitations. Nasopharyngeal swabs can be uncomfortable and even painful for patients, which can lead to reluctance to get tested [6]. Moreover, the need for trained healthcare professionals to perform these invasive procedures can add additional strain to healthcare systems that are already overwhelmed with the demands of the pandemic [7]. Sample collection and processing challenges can also lead to delayed results due to laboratory processing times [28].

### 2.3. Overview of Current Non-Invasive Testing Methods

Non-invasive methods of SARS-CoV-2 testing have the potential to address these challenges and improve testing accessibility. One of the most promising non-invasive testing methods is saliva-based testing. Saliva-based testing involves collecting a sample of the patient’s saliva, which is then analyzed for the presence of SARS-CoV-2. This method is less invasive than nasopharyngeal swabs and can be self-collected, reducing the need for trained healthcare professionals to perform testing procedures. Except for being more convenient and less painful for patients, saliva-based testing is cost-effective and scalable, as it does not require specialized equipment or expertise for collection and processing [10].

Non-invasive testing methods, such as saliva-based testing, gained attention due to their ease of use and non-invasive nature. However, one of the main limitations of saliva-based testing is the lower concentration of SARS-CoV-2 viral load in saliva compared to nasopharyngeal swabs, which can lead to false negatives if the testing device is not sufficiently sensitive [29,30]. While biosensors have the potential to provide highly sensitive and specific detection of viral particles, even in low concentrations, they may also be affected by measurement noise, which can lead to inaccurate results, particularly at low viral loads [31,32,33].

To overcome this challenge, machine learning-enhanced biosensors are being developed. Machine learning algorithms can help to distinguish signal from noise in the biosensor measurements, improving the accuracy of SARS-CoV-2 detection even at low viral loads. This technology has the potential to revolutionize SARS-CoV-2 testing, allowing for more accurate and accessible non-invasive testing methods.

## 3. Machine Learning-Enhanced Biosensors for Non-Invasive Sampling

### 3.1. Machine Learning-Enhanced Biosensors

**Figure 1:**
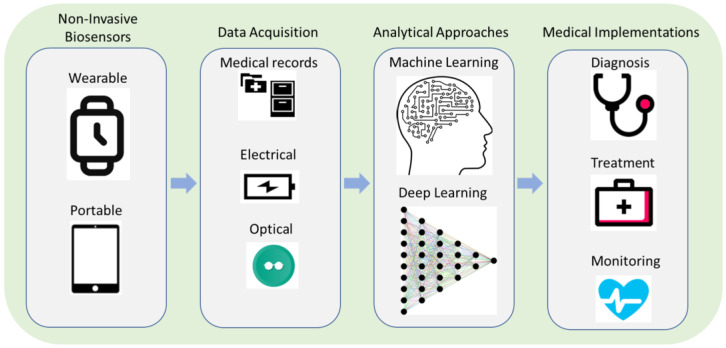
Non-invasive biosensors are enhanced with ML algorithms for medical applications.

Machine learning-enhanced biosensors represent a promising approach to improve the sensitivity and specificity of non-invasive SARS-CoV-2 testing. Biosensors are analytical devices that can detect and quantify biological molecules, such as viral RNA or antigens, in a sample. However, several factors, including sample quality, background noise, and other sources of interference, may limit the accuracy and sensitivity of biosensors. As shown in Figure 1, machine learning algorithms can be integrated with biosensors to enhance their performance by analyzing large amounts of data and identifying patterns that can be used to improve the accuracy and sensitivity of the biosensor [15,34].

### 3.2. Pattern Recognition and Error Detection

**Figure 2:**
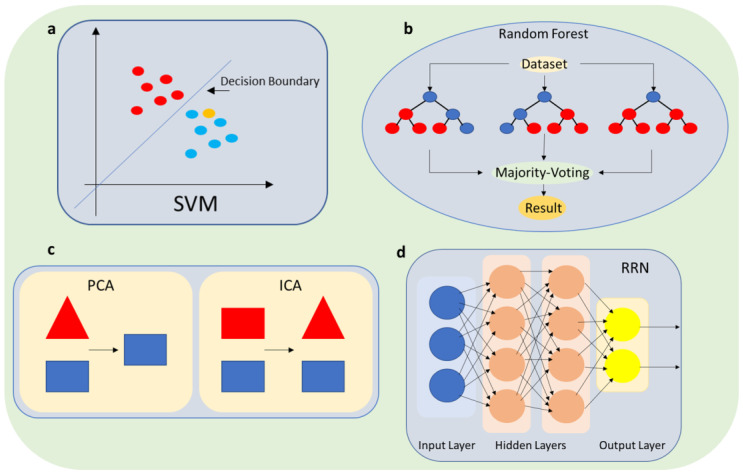
Schematic illustration of ML algorithms; (**a**) SVM; (**b**) random forest; (**c**) PCA-ICA; and (**d**) RRN.

To overcome the limitations of biosensors in detecting low concentrations of SARS-CoV-2 in saliva samples, machine learning algorithms can be used to perform automated quality control of biosensor measurements. By analyzing large datasets, machine learning algorithms can identify patterns in the signal that correspond to the presence of the target analyte (in this case, SARS-CoV-2). Once these patterns are identified, the biosensor can use them to detect the presence of the virus more accurately in saliva samples. Employing supervised learning algorithms, such as support vector machines (SVMs) [35] (Figure 2a) or random forests [36] (Figure 2b), is one way to make use of these patterns. These algorithms can classify each measurement as either accurate or erroneous after being trained on a sizable dataset of biosensor readings with known ground truth values. The algorithm learns the fundamental patterns and relationships in the data throughout this training process, which enables it to predict new measurements with accuracy. The accuracy of the test results can be increased by highlighting measurements that the algorithm finds to be inaccurate. This strategy has the potential to completely transform the field of biosensing and showed considerable promise in terms of enhancing the precision and dependability of biosensors, notably in the context of non-invasive SARS-CoV-2 testing. Specific examples will be given in Section 4 of using supervised algorithms such as SVMs to improve SARS-CoV-2 biosensors by recognizing specific patterns in a dataset and assigning the data to the correct class [37,38].

**Figure 3:**
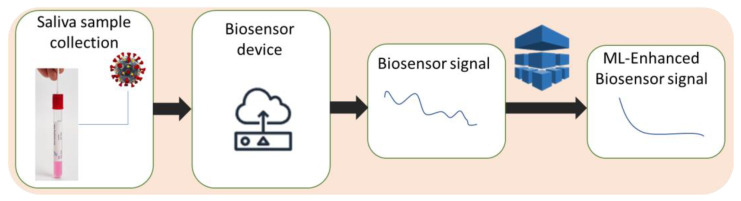
Machine learning algorithms improve biosensor signal by distinguishing it from measurement noise.

Moreover, machine learning can also enable biosensors to detect errors or noise in the measurement process. Noise in the data can arise from various sources, such as variations in the sample matrix or environmental factors such as temperature and humidity. By analyzing patterns in the data and comparing them to known signal patterns, machine learning algorithms can distinguish signal from noise and reduce false positives and false negatives in the measurements, as shown in Figure 3. Specifically, unsupervised learning algorithms such as principal component analysis (PCA) [39,40] or independent component analysis (ICA) [41] (Figure 2c) can be used to identify patterns and correlations in the biosensor measurements and separate them from noise. Experimental results [42,43] show that by using these algorithms, the biosensor can better discriminate between the signal from the virus and other noise sources, such as background fluorescence in the case of sensitive detection of porcine epidemic diarrhea virus [42], or even facilitate the classification of viruses based solely on their intrinsic spectra [43], as well as improve the sensitivity and specificity of the test. PCA was also used successfully to enhance the biomarker selection process and improve the performance of the classifier in distinguishing between different classes of proteins and viruses [44].

Thus, machine learning-enhanced biosensors can provide a more accurate and reliable non-invasive method for detecting SARS-CoV-2 in saliva samples. The ability to detect low concentrations of the virus with high specificity and sensitivity could greatly improve the accessibility and effectiveness of testing efforts, particularly in areas where invasive testing methods are not readily available or feasible.

### 3.3. Contrastive Learning

Contrastive learning is a powerful technique used in machine learning to help models learn to distinguish between different types of data points [14]. The main goal of contrastive learning is to create pairs of data points that are similar in some way (positive pairs) and pairs that are dissimilar (negative pairs). By training models to distinguish between these two types of pairs, we can promote the closeness of similar pair representations and increase the orthogonality of dissimilar pair representations [45]. Contrastive learning can be applied to biosensors to learn data representations that highlight the distinctions between various signal kinds [46]. This is advantageous for biosensors since the signals they pick up are frequently rather weak and are easily masked by background noise or other forms of interference. Contrastive learning can help to increase the accuracy of biosensors by making it simpler to discern between various types of signals by teaching representations that highlight the contrasts between signals.

Contrastive learning can be a useful strategy for learning meaningful representations that highlight the differences between various signal types in the setting of biosensors, such as cardiac signals [47], electroencephalogram (EEG), and electrocardiogram (ECG) [48,49]. Weak signals that are easily obscured by background noise or other types of interference are common characteristics of biosignals. By using contrastive learning, the models can be trained to distinguish between various signal types based on their intrinsic contrasts, improving the accuracy of biosensors.

The unique difficulties posed in this field must be carefully taken into account when using contrastive learning for biosensors. Biosignal datasets frequently feature a small number of subjects—generally under 100—and noisy labeling. Cheng et al. suggested that a self-supervised approach based on contrastive learning can be modified to overcome these difficulties [49]. This strategy seeks to simulate biosignals with a smaller number of patients and less reliance on labeled data.

Intersubject variability can have a detrimental effect on model performance in the regime of restricted labels and individuals. Subject-aware learning methods can be used to lessen this problem. One method is to use a subject-specific contrastive loss, where the contrastive loss is calculated using distributions specific to the person. This motivates the model to discover representations that accurately reflect the distinctive features of each subject’s biosignals.

Additionally, subject invariance can be encouraged during the self-supervised learning process by using adversarial training. The model’s ability to generalize across diverse subjects and perform better as a whole can be improved by training it to be invariant to subject-specific variances.

**Figure 4:**
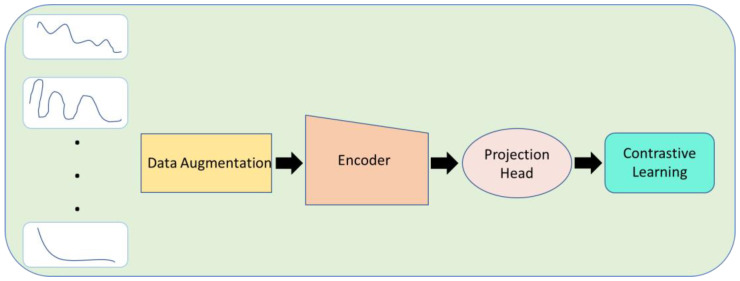
Contrastive learning highlights distinctions between various signals.

Figure 4 depicts a comprehensive pipeline for applying unsupervised/contrastive learning in the context of biosensors.

Data augmentation is the first process in the pipeline, starting with the biosensor signals as the input layer. To increase the diversity and robustness of the training dataset, this method entails producing extra varieties of the input data. Techniques for enhancing data may involve random translations, rotations, noise addition, or additional modifications.

The signals are then passed to an encoder after data augmentation. The encoder, which learns to extract meaningful and condensed representations from the input data, is a crucial component. It captures crucial features and patterns by transforming the high-dimensional signals into a lower-dimensional latent space.

The encoded representations are then supplied onto a projection head after the encoder. The separability and discriminative capabilities of the encoded representations are improved by the projection head’s additional mapping of the latent space to a separate feature space. The projection head may include one or more layers that subject the latent features to different non-linear transformations.

The pipeline culminates in contrastive learning, a self-supervised learning method that tries to reduce the agreement between dissimilar cases while increasing the agreement between comparable instances. The model learns to combine the encoded representations of similar biosensor signals and push apart the representations of dissimilar signals by utilizing contrastive learning. Through this procedure, the model is urged to acquire accurate representations of the underlying structure and properties of the biosensor data.

### 3.4. Real-Time Interpretation of Biosensor Measurements with Machine Learning

With the integration of machine learning (ML) techniques, biosensors can provide real-time interpretation of test results even after a few initial measurements, therefore providing a SARS-CoV-2 result in few seconds.

ML algorithms can be effectively trained to discern the specific signature of SARS-CoV-2 in saliva samples, effectively separating it from background noise and other interferences. Signal processing techniques play a crucial role in this process, with mathematical models such as recurrent neural networks (RNNs) (Figure 2d) being employed to extract meaningful information from biosensor signals [50]. Among these techniques, wavelet transform stands out as a valuable approach, enabling the decomposition of the biosensor signal into different frequencies and aiding in the discrimination between signal and noise [51]. RNNs excel in biosensor signal processing tasks due to their ability to capture intricate temporal dependencies within the data. The decomposition of the biosensor signal into distinct frequency bands, a common strategy in biosensing, enhances the differentiation between signal and noise. Leveraging the power of wavelet transform and other signal processing methods, machine learning algorithms can successfully extract valuable insights from biosensor signals, leading to more accurate and sensitive detection of SARS-CoV-2 in saliva samples.

Another proposed method that might significantly boost the effectiveness and precision of biosensor testing is adaptive sampling [52,53]. Based on the parameters of the saliva sample being examined, this method entails varying the sampling rate and length. For instance, the algorithm may lower the sample frequency and length if the biosensor identifies a strong signal from the early measurements because it is likely that the signal will stay high throughout the test. On the other hand, if the first measurements reveal a weak or noisy signal, the algorithm may raise the sample frequency and length to gather more data and improve the accuracy of the outcome [54,55]. Compared to conventional testing techniques, which generally include a predetermined sample rate and length, adaptive sampling provides several advantages. Adaptive sampling can decrease the time needed for accurate detection while simultaneously boosting the dependability of the result by modifying the sampling rate and duration based on the features of the sample. This is especially vital for applications that need quick and precise detection, including clinical diagnostics or environmental monitoring. By examining the characteristics of the saliva sample and modifying the sampling rate and length appropriately, machine learning algorithms may be trained to accomplish adaptive sampling. A sizable dataset of biosensor readings with well-known ground truth values is necessary for this approach in order to train the algorithm and enhance its performance. Once it is trained, the algorithm may be connected with the biosensor to deliver real-time adaptive sampling and boost testing precision and effectiveness.

## 4. Applications of Machine Learning-Enhanced Biosensors for SARS-CoV-2 Testing

Up to this point, various biosensor-based approaches were developed for the accurate detection of SARS-CoV-2 and monitoring of its spread. However, only a few of these approaches were enhanced with machine learning algorithms to improve their functionality.

Beduk et al. [56] developed a tiny machine learning (TinyML) algorithm that can detect SARS-CoV-2 variants in patient samples using a PoC biosensor device called KAUSTat. TinyML refers to the development of machine learning algorithms that can run on low-power microcontrollers with limited resources, such as memory and computing power. This enables the integration of machine learning capabilities into small and portable devices, such as the KAUSTat biosensor, which can then perform complex tasks without the need for large and expensive computing equipment. The algorithm was trained on a dataset of 4224 differential pulse voltammetry (DPV) curves and achieved high accuracy in identifying Beta, Alpha, and Delta variants, as well as control patients. The dataset was used to train a deep neural network (DNN). The DNN was able to achieve a high level of accuracy, resulting in 98.7% accuracy in inferring Beta variant, 99.5% accuracy in inferring Alpha variant, while 100% accuracy was obtained in inferring Delta and 99.37% accuracy in control patients. The total combined accuracy of the proposed neural algorithm was 99.37%. The KAUSTat device takes around 1 min to read the prepared sensor after patient sample incubation for 2 h and approximately 20 ms to define the patient as positive or negative and identify the specific type of variant [56].

In another study, machine learning (ML) techniques were used to process the signal output of a SARS-CoV-2 immunosensor to classify samples as positive or negative and improve the accuracy of the measurement. The ML techniques used were support vector machine (SVM) classifiers with different kernel functions evaluated using the MATLAB Classification Learner app. A set of 55 positive and 53 negative samples were used for training and validation purposes. The results show that the linear, quadratic, and cubic kernels performed well, but the linear kernel was chosen for future embedded implementation and C code generation due to its greater simplicity. The ML algorithm was able to recognize specific patterns within the provided dataset and classify unknown data, improving test accuracy to 97.3%. The ML approach was found to have better performance and allowed baseline drift to be automatically overcome, thus simplifying the data preprocessing during the testing phase. The ML algorithm can also be easily integrated into the cloud-based portable Wi-Fi device [37].

Deep learning is used as well for SARS-CoV-2 detection. For example, deep learning-based CT diagnosis is a method of medical diagnosis that utilizes deep learning algorithms to analyze medical images such as typically computed tomography (CT) scans. With this approach, deep neural networks are trained on large sets of labeled medical images, which enables them to learn patterns and features that are associated with specific diseases or conditions. Once trained, these models can be used to analyze new CT scans and provide diagnostic insights to physicians [13,14,57].

However, deep learning is also used on SARS-CoV-2 biosensors. Potter et al. developed an optical biosensor that collects data in the form of images of individual microbeads undergoing agglutination. These images are obtained using a portable in-line lens-free holographic microscope and are analyzed by a deep learning algorithm that distinguishes microbead agglutination from cell debris and viral particle aggregates. The agglutination is then quantified based on the network output, which allows for the determination of viral concentrations. A deep convolutional neural network (CNN) with residual connections was used to analyze the images. The network was designed to handle the complex imaging conditions present in the SARS-CoV-2 agglutination assay. They used a 4-block-deep CNN with residual connections and trained it using a single intensity threshold to identify features of interest in several agglutination assay images. The network was hand-classified into 5 categories and 11,280 images were used. A total of 75% of these images were used for training, and 25% were reserved for validation. Validation was performed every 2 epochs, and training was halted after the classification accuracy of the validation image set stopped improving to prevent overtraining. The final validation accuracy of the trained CNN was 82.06% [58].

In other research, a method was proposed to detect symptomatic COVID-19 patients using heart rate variability (HRV) signals. A deep learning technique called a convolutional autoencoder (CAE) was used to learn the features of the HRV signals and then classify them into symptomatic or asymptomatic classes. The heart rate data were remotely collected using a wearable biosensor. A CAE is a type of neural network that consists of two parts: an encoder and a decoder. The encoder compresses the input signal into a lower-dimensional representation, and the decoder tries to reconstruct the original input signal from this representation. By minimizing the difference between the original and reconstructed signals, the CAE can learn meaningful features of the input data. The authors used a contrastive loss function to train the CAE. The contrastive loss function aims to minimize the difference between the reconstruction errors of the symptomatic and asymptomatic signals. This way, the CAE can learn to distinguish between symptomatic and asymptomatic signals and produce lower reconstruction errors for asymptomatic signals and higher reconstruction errors for symptomatic signals. The contrastive CAE outperforms a conventional convolutional neural network (CNN), a long short-term memory (LSTM) model, and a convolutional auto-encoder without contrastive loss (CAE). Overall, the proposed method achieved high accuracy in detecting symptomatic COVID-19 patients using HRV signals [59].

Finally, ML and deep learning (DL) approaches were used for detecting SARS-CoV-2 using an electrochemical sensor called UFC-19. The authors tested virus samples including SARS-CoV, MERS-CoV, human CoV, and influenza using UFC-19 and obtained a current response dataset. They then used different algorithms to diagnose SARS-CoV-2 from this dataset and found that the convolution neural networks algorithm was able to diagnose SARS-CoV-2 samples with high sensitivity, specificity, and accuracy. For ML algorithms, they used four different models: support vector machine (SVM), random forest (RF), AdaBoost classifier (ABC), and decision tree classifier (DTC). They fed different feature combinations to each of these algorithms and tested them 25 times to determine their accuracy and standard deviation. The best accuracy (96.6%) was achieved by DTC. Additionally, they used a convolutional neural network (CNN) to optimize the classification results. They tried different layer numbers, activation functions, filter sizes, neuron numbers, etc., and optimized the model parameters to achieve the highest repeatable accuracy, sensitivity, and specificity. The highest achieved accuracy was 97.2%, which outperformed the DTC algorithm. They suggested that combining this CNN model with UFC-19 could enable selective identification of SARS-CoV-2 presence within two minutes [38].

A brief summary of ML-enhanced biosensors for SARS-CoV-2 detection is shown in Table 1.

## 5. Conclusions and Future Directions

With the ongoing COVID-19 pandemic, it is crucial for a large part of the population to undergo COVID-19 testing and medical screening. Testing and screening are essential to detect and contain the spread of the virus, especially in asymptomatic individuals who can unknowingly transmit the virus to others. Non-invasive sampling methods, such as saliva, were found to be less uncomfortable and invasive than traditional methods, such as nasopharyngeal swabs. This reduces patient anxiety and discomfort during the test, making it easier for a larger portion of the population to participate in the testing process. In order to use such sampling methods though, there need to be testing methods that are sensitive enough to efficiently detect SARS-CoV-2 presence in saliva samples. Various types of biosensors were developed for this purpose. Machine learning algorithms can enhance these biosensors in order to crucially improve their sensitivity.

There are several limitations and challenges associated with the use of machine learning-enhanced biosensors for SARS-CoV-2 testing. Even though after their development these devices are usually much simpler and easier to use for virus detection than traditional testing methods, there is a need for specialized equipment and expertise for their development. More importantly, there is a need for large amounts of high-quality data to train machine learning algorithms. Low-quality or biased data can negatively impact the performance and reliability of the biosensors [60,61]. Moreover, as is the case with every application that uses the internet, there is the issue of data privacy. ML-enhanced biosensor systems should contain tools and instructions in order to avoid leaking users’ medical data. The use of such systems should not be the only facet under ethical consideration. Additionally, the design of ML-enhanced biosensor systems is crucial in order to avoid the creation of one more technology that could be characterized as a weapon of math destruction [62].

An additional challenge that should be considered when designing ML-enhanced biosensors is interpretability. ML algorithms are often considered “black boxes” with limited interpretability [63,64]. Especially in medical applications, where understanding the reasoning behind a decision is critical, the challenge of interpretability should be addressed, as it directly impacts the adoption and trust of ML-enhanced biosensors in clinical settings, and it enhances transparency and aids in error detection [65].

Despite these challenges, ML-enhanced biosensors represent a promising approach to improve the accuracy, speed, and accessibility of SARS-CoV-2 testing. Future research in this field should focus on optimizing biosensor design and performance, as well as developing robust and user-friendly biosensor platforms. Furthermore, addressing scalability concerns and exploring the potential of cloud computing for data processing could significantly enhance the practical applicability of ML-enhanced biosensors for widespread testing. The development of secure and efficient data sharing mechanisms can facilitate access to high-quality datasets for training ML models effectively.

Contrastive learning especially emerges as a promising approach for analyzing biosignals. Its effectiveness in extracting informative patterns from noisy signals, with minimal preprocessing requirements, suggests its potential for enhancing biosensor applications. The utilization of self-supervised learning, specifically contrastive learning, opens new possibilities for personalized healthcare and the application of machine learning algorithms in practical healthcare settings. Moreover, the integration of machine learning algorithms with biosensors can be extended to other areas of healthcare, including disease diagnosis and drug discovery. By continually exploring and adopting cutting-edge ML techniques, the field of machine learning-enhanced biosensors for SARS-CoV-2 testing will continue to progress, leading to improved testing accuracy, faster results, and enhanced accessibility. Embracing an interdisciplinary approach that encompasses expertise from biosensor engineering, ML, data privacy, and healthcare will be essential to overcome the remaining challenges and realize the full potential of ML-enhanced biosensors for effective SARS-CoV-2 testing and beyond.

## Figures and Tables

**Table 1:** ML-enhanced biosensors for SARS-CoV-2 detection.

Biomarker	Mechanism	Forms of Collected Data	ML Algorithm	Ref
Spike protein	Electrical	Current	DNN	[56]
Spike protein	Electrical	Current	SVM	[37]
SARS-CoV-2 pseudovirus	Optical	Images	CNN	[58]
Heart rate	Optical	HRV signals	Contrastive CAE	[59]
Spike protein	Electrical	Current	SVM, RF, ABC, DTC, CNN	[38]
